# Prospective motion correction of 3D echo-planar imaging data for functional MRI using optical tracking

**DOI:** 10.1016/j.neuroimage.2015.03.013

**Published:** 2015-06

**Authors:** Nick Todd, Oliver Josephs, Martina F. Callaghan, Antoine Lutti, Nikolaus Weiskopf

**Affiliations:** a Wellcome Trust Centre for Neuroimaging, UCL Institute of Neurology, University College London, London, UK; b Laboratoire de Recherche en Neuroimagerie, Department of Clinical Neurosciences, Centre Hospitalier Universitaire Vaudois, Lausanne, Switzerland

**Keywords:** Prospective motion correction, Functional MRI, 3D imaging, EPI, Optical tracking

## Abstract

We evaluated the performance of an optical camera based prospective motion correction (PMC) system in improving the quality of 3D echo-planar imaging functional MRI data. An optical camera and external marker were used to dynamically track the head movement of subjects during fMRI scanning. PMC was performed by using the motion information to dynamically update the sequence's RF excitation and gradient waveforms such that the field-of-view was realigned to match the subject's head movement. Task-free fMRI experiments on five healthy volunteers followed a 2 × 2 × 3 factorial design with the following factors: PMC on or off; 3.0 mm or 1.5 mm isotropic resolution; and no, slow, or fast head movements. Visual and motor fMRI experiments were additionally performed on one of the volunteers at 1.5 mm resolution comparing PMC on vs PMC off for no and slow head movements. Metrics were developed to quantify the amount of motion as it occurred relative to k-space data acquisition. The motion quantification metric collapsed the very rich camera tracking data into one scalar value for each image volume that was strongly predictive of motion-induced artifacts. The PMC system did not introduce extraneous artifacts for the no motion conditions and improved the time series temporal signal-to-noise by 30% to 40% for all combinations of low/high resolution and slow/fast head movement relative to the standard acquisition with no prospective correction. The numbers of activated voxels (p < 0.001, uncorrected) in both task-based experiments were comparable for the no motion cases and increased by 78% and 330%, respectively, for PMC on versus PMC off in the slow motion cases. The PMC system is a robust solution to decrease the motion sensitivity of multi-shot 3D EPI sequences and thereby overcome one of the main roadblocks to their widespread use in fMRI studies.

## Highlights

• Data quality was assessed in terms of tSNR, RMSE, and task fMRI t-statistics.
• Correction improved tSNR by 30% to 40%.
• Percent reduction in RMSE as a function of motion level was characterized.
• Number of active voxels for motor and visual tasks increased significantly.

## Introduction

Subject motion in functional magnetic resonance imaging (fMRI) is the most important cause of temporal instability in the data. Typically, fMRI studies attempt to detect changes from the mean MR signal on the order of 1% and therefore even minor degradation of the data due to motion can mask effects of interest. When motion occurs during an fMRI time series, it is most commonly handled with post-processing methods that assume rigid body motion and attempt to co-register and align all image volumes within the time series (Ashburner and Friston, 2003). These methods are effective for correcting slow inter-volume motion, but cannot handle the problem of faster intra-volume motion. The more serious problem of intra-volume motion is most often managed by a combination of restraining the head as much as possible (Yacoub et al., 2008) and scanning with a 2D echo-planar imaging (EPI) sequence that acquires a single slice within typically 20–80 ms, thereby “freezing” most subject motion (Feinberg and Yacoub, 2012). However, even these approaches do not solve the motion problem completely as overly rigid head immobilization is not always practical depending on the subject's level of comfort and the functional task being performed. In addition, motion between slice acquisitions cannot be corrected for by the most frequently used fMRI analysis packages (e.g., Friston et al., 2007; Goebel et al., 2006; Smith et al., 2004; SPM8) and a multi-slice 2D volume can still be corrupted by spin history related artifacts and motion in the through-plane direction.

While single-shot 2D EPI sequences remain the dominant method for data acquisition in fMRI studies, researchers are investigating both 2D multi-band EPI and 3D EPI sequences for improved performance. 2D multi-band EPI sequences offer improved temporal resolution by accelerating the data acquisition along the slice direction while remaining a single-shot method (Setsompop et al., 2012). The main advantage of 3D EPI imaging is the signal-to-noise (SNR) gain achieved by volumetric excitation. The slab selective excitation and 3D Fourier encoding used in 3D imaging mean that the amount of data contributing to the k-space signal is increased by a factor equal to the number of slices acquired, N_slices_, which leads to a theoretical SNR improvement proportional to √N_slices_ compared to the same volume acquired with a multi-slice 2D excitation and readout (Hu and Glover, 2007; Poser et al., 2010). This theoretical gain in SNR for 3D EPI compared to 2D EPI sequences is realized in practice when imaging at high resolution as the data are in a regime dominated by thermal noise over physiological noise (Hu and Glover, 2007; Lutti et al., 2013; Poser et al., 2010; van der Zwaag et al., 2012). Several recent studies have shown that this improved SNR at high resolution translated into superior BOLD sensitivity at 3 T (Lutti et al., 2013) and 7 T (Jorge et al., 2013; Poser et al., 2010; van der Zwaag et al., 2012). Further advantages of 3D imaging at higher resolutions include the ability to simultaneously under-sample the k-space data in both phase-encoding directions, and thereby realize greater data acquisition acceleration factors (Poser et al., 2010), reduced specific absorption rate (SAR), less demanding k_z_ gradient requirements, and avoidance of slice profile imperfections that occur in 2D imaging (Poser et al., 2010; Tijssen et al., 2011; van der Zwaag et al., 2012).

Despite these advantages, when it comes to handling subject motion multi-shot 3D EPI sequences have the crucial drawback of acquiring data for image formation over a time scale of several hundred milliseconds to several seconds instead of tens of milliseconds. Post-processing methods for realigning image volumes of 3D EPI data can still correct inter-volume motion, but intra-volume motion occurring at any time during the 3D acquisition will corrupt the whole image volume and not only a few slices as in single-shot 2D EPI. Strategies for correcting MR data with intra-volume motion can be broadly classified into approaches based on how the motion is estimated (internal MR data vs external sensors), assumptions about the type of motion (affine vs deformable), and when the correction is done (retrospective vs prospective). Approaches that use internal data to estimate motion, such as k-space-based or image-based navigators, need additional sequence time to acquire that information, but the demanding requirements in fMRI studies of high spatiotemporal resolution and whole brain volume coverage necessitate that the data sampling be as efficient as possible. The assumption that head movements can be characterized as rigid body translations and rotations is a very good approximation for all areas above the intercommissural (AC–PC) line, and therefore more complicated approaches to motion correction that can account for deformations (Atkinson et al., 1997; Hutton et al., 2013) are not necessary (Ashburner and Friston, 2003). Retrospective approaches can be very powerful but they all have one or more limitations for the current application, including the need to operate in the k-space domain, potentially lengthy computation time, no indication of image quality until after the scanning session is over, and the potential for sub-Nyquist k-space sampling when large head rotations occur.

Here we present an approach to correct motion during 3D EPI scanning for fMRI applications. In light of the constraints noted above, we have chosen a method that uses an external sensor, assumes rigid body motion, and is applied prospectively. The prospective motion correction (PMC) system uses an optical camera to track a marker that is rigidly attached to the subject's head. The motion information is used to realign the imaging field-of-view (FOV) with the subject's measured head position and orientation immediately before each radio-frequency (RF) pulse. The performance of the approach was tested on five healthy volunteers during task-free fMRI scanning, with the following conditions in a 2 × 2 × 3 factorial design: PMC on vs off; 3.0 mm isotropic vs 1.5 mm isotropic spatial resolution; no head movement, intentional slow head movement, and intentional fast head movement. Further testing was done on one of the volunteers during visual and motor task fMRI using a 2 × 2 factorial design of PMC on vs PMC off and no head movement vs head movement. The objective of the study was to evaluate the extent to which the PMC system can improve the quality of the 3D EPI data under these various conditions.

## Methods

### PMC camera and sequence control system

The PMC system uses an optical camera (Kineticor, HI, USA) mounted on the inside of the scanner bore to track the motion of a passive Moire phase marker at 80 Hz frame rate (Maclaren et al., 2012). Grating patterns and fixed markings on the Moire phase marker allow the three translational and three rotational degrees of freedom to be measured with precision on the order of tens of microns for the translations and hundredths of degrees for the rotations (Maclaren et al., 2012). The information containing the position and orientation of the marker is sent to the scanner host computer, where the data are transformed from camera coordinates to scanner coordinates using a pre-calibrated transformation matrix. Special libraries have been developed in the Siemens IDEA programming environment (version VB17) to use this data to dynamically update the imaging FOV such that it follows the movement of the marker (Herbst et al., 2012, 2014; Speck et al., 2006; Zaitsev et al., 2006). This is done immediately before each excitation by updating the RF pulse frequency, the RF pulse phase offset, and the amplitudes of the imaging gradients. The camera time-series data are not temporally smoothed or filtered before being sent to the MR host. The motion information that is logged by the PMC system gives the three translation and three rotation measurements of the marker relative to its initial position.

### 3D EPI sequence

The 3D EPI sequence acquired each k_x_–k_y_ plane of data in one echo train using Cartesian sampling and applied linearly ascending phase encoding along the partition dimension, where partition refers to the slow phase encoding direction (Lutti et al., 2013). The experimental design was set up to test the PMC system at two different resolutions, 3.0 mm isotropic and 1.5 mm isotropic. Sequence parameters were kept as similar as possible between the two different resolutions. The two versions of the sequence had different imaging matrices (64 × 64 × 44 vs 128 × 128 × 44, giving 44 RF excitations per volume for both versions), different bandwidths (2367 Hz/pixel vs 1395 Hz/pixel), and the high-resolution acquisition was accelerated by GRAPPA parallel imaging with factor 2 in the phase encoding direction. All other parameters were as follows for both sequences: Transverse slice orientation with phase encoding along the anterior–posterior direction, repetition time TR = 78 ms, echo time TE = 37.3 ms, flip angle = 15°, 10% data oversampling in the partition direction, 3.4 second acquisition time per image volume.

### Imaging experiments

**Fig. 1:**
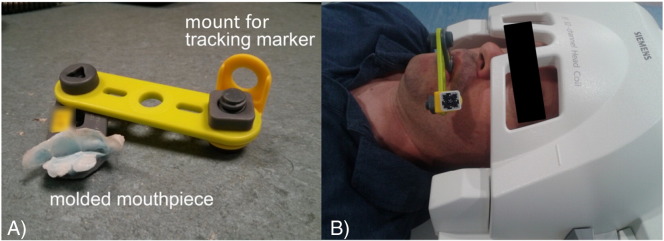
Mini bite bar set up used to securely attach the tracking marker to the subject. A) Each bite bar was individually molded to the volunteer's upper front teeth using a medical grade hydroplastic (TAK Systems) and included a two-hinge mounting system for flexible placement of the tracking marker. B) Image of a volunteer with the mini bite bar and tracking marker taken from approximately the same angle as where the camera would be located in the scanner bore.

All scanning was performed on a Siemens TIM Trio scanner using a standard 32-channel head coil and with volunteer informed consent obtained under the approval of the institution's local ethics committee. Five healthy volunteers were scanned under task-free fMRI conditions and one of the volunteers was also scanned in two separate task fMRI sessions. Custom made mini bite bars were used to rigidly attach the Moire phase marker to the front upper teeth of the volunteer, thereby ensuring the best coupling of marker and head movement (see Fig. 1).

To fully test the performance of the PMC system, the task-free imaging experiments were set up as a 2 × 2 × 3 factorial design, resulting in twelve permutations of the following conditions: PMC on vs PMC off; 3.0 mm isotropic resolution vs 1.5 mm isotropic resolution; and no head movement vs slow head movement vs fast head movement. For each combination of conditions, 100 image volumes were acquired over 5 min and 40 s. The volunteers were told which type of head movement to do before the start of each run, but were blinded regarding the sequence resolution and whether PMC was on or off. The order of the twelve conditions was randomized over the volunteers. For the cases of slow and fast head movement, the volunteers were instructed to keep still for the first 12 image volumes in order to obtain artifact-free data to be used in the creation of a reference image (described below). Motion data were recorded for use in analysis in all conditions. This information was used to prospectively correct the FOV alignment for the PMC on condition.

The type of motion that the volunteers were instructed to carry out was designed to sample as well as possible the full range of motions seen in fMRI studies, from compliant healthy volunteers to more difficult patient populations (Brown et al., 2010; Lemieux et al., 2007; Van Dijk et al., 2012). Before scanning, volunteers were coached to limit the amplitude of their translational and rotational movements such that the marker movement stayed within a range of ± 10 mm and ± 5°. This was done by projecting the real-time camera motion traces onto a screen in the scanner bore for the volunteer to see. The volunteers were instructed to intersperse periods of movement with periods of lying still such that different image volumes would have differing amounts of total motion occurring during the data acquisition period, and so that the motion would occur at different times with respect to the partition encoding. The movement events were frequent compared to what would typically be seen from a compliant volunteer, but not beyond the upper limit of what has been reported for either healthy volunteers (Van Dijk et al., 2012) or patients (Brown et al., 2010; Lemieux et al., 2007). The relatively high rate of movement events was deliberately chosen in order to efficiently sample as much of the motion trajectory space as possible.

For the task-based imaging experiments, the fMRI time series data were acquired with the 3D-EPI sequence at 1.5 mm isotropic resolution. The 2 × 2 factorial designed consisted of conditions of PMC on vs PMC off and no head movement vs slow head movement. The visual paradigm used a 10-Hz flickering black/white checkerboard alternately stimulating the left and right visual hemifields. The motor paradigm was a standard finger-to-thumb tapping task where the volunteer alternated blocks of tapping with the left and right hands. For both tasks, block designs of 15-second left stimulation, 15-second rest, 15-second right stimulation, 15-second rest were repeated 5 times for a total scan time of 5 min.

### Data analysis

The goal of the study was to evaluate the performance of the PMC system as a function of head movement. In order to accomplish this, several metrics were established to quantify both the amount of motion occurring during data acquisition and the image quality resulting from the acquired data.

The first motion metric, *total speed*, combined the high temporal resolution camera-measured translations (in mm), x, y, z, and rotations (in degrees), P, R, Y, into a single scalar quantity, *S*, designed to measure the rate of the motion:

(1) $$S=\sqrt{{\left(\frac{dx}{dt}\right)}^{2}+{\left(\frac{dy}{dt}\right)}^{2}+{\left(\frac{dz}{dt}\right)}^{2}+{\left(\frac{dP}{dt}\right)}^{2}+{\left(\frac{dR}{dt}\right)}^{2}+{\left(\frac{dY}{dt}\right)}^{2}}\text{.}$$

*S* has the temporal resolution of the camera data. To remove noise due to mechanical scanner vibration and other sources, a low pass filter was applied to the translation and rotation data to remove information above 10 Hz (the scanner vibration noise occurred at the partition repetition frequency, 12.8 Hz). Note that weighting the rotations by the same amount as the translations is equivalent to assuming a rotational radius of 5.7 cm, which is reasonable considering the typical head size.

The next two motion metrics, *integrated motion* and *partition-weighted integrated motion*, were designed to create a single estimate of the total amount of motion that occurred throughout the acquisition of one image volume. The integrated motion metric is a simple numerical integration of the total speed metric over the time of each image volume.

(2) $$M=\sum_{i}\left(S_{i}\cdot \Delta t\right)$$

*S_i_* refers to the *i*th data point of the total speed variable, Δ*t* is the time step between camera samples, and the sum is over all data points within one image volume acquisition. *M* has the temporal resolution of the image volume acquisition rate and the total speed values are all weighted equally. The next metric, *partition-weighted integrated motion*, is designed to reflect the fact that the k-space energy is distributed non-uniformly along the partition encoding dimension, and therefore motion occurring during different partition encoding steps will affect the image quality differently. The partition weightings are determined by converting the reference image of the time series into k-space using a Fourier transform and integrating the squared modulus of the k-space values, *c*, over the *j*th k_x_–k_y_ plane:

(3) $$k_{j}=\sqrt{\sum_{kx_{j}}\sum_{ky_{j}}\left(c\left(kx_{j},ky_{j}\right)\cdot c{\left(kx_{j},ky_{j}\right)}^{*}\right)}\text{.}$$

These weightings for the *j*th partition are then used to create the *partition-weighted integrated motion* metric:

(4) $$M_{PW}=\sum_{j}\sum_{i}\left(S_{i,j}\cdot k_{j}\cdot \Delta t\right)\text{.}$$

Here the sums are now over the *i* data points of the total speed variable that are within a single partition and over the *j* partitions that make up the image volume. Note that because the Fourier transform of the reference image was done on the magnitude of the image, an artificial symmetry is imposed on the k-space that may not have existed in the original complex multi-channel data. This will affect the *k_j_* values by a few percent, but was deemed preferable to calculating the weights from the large raw k-space data sets.

**Fig. 2:**
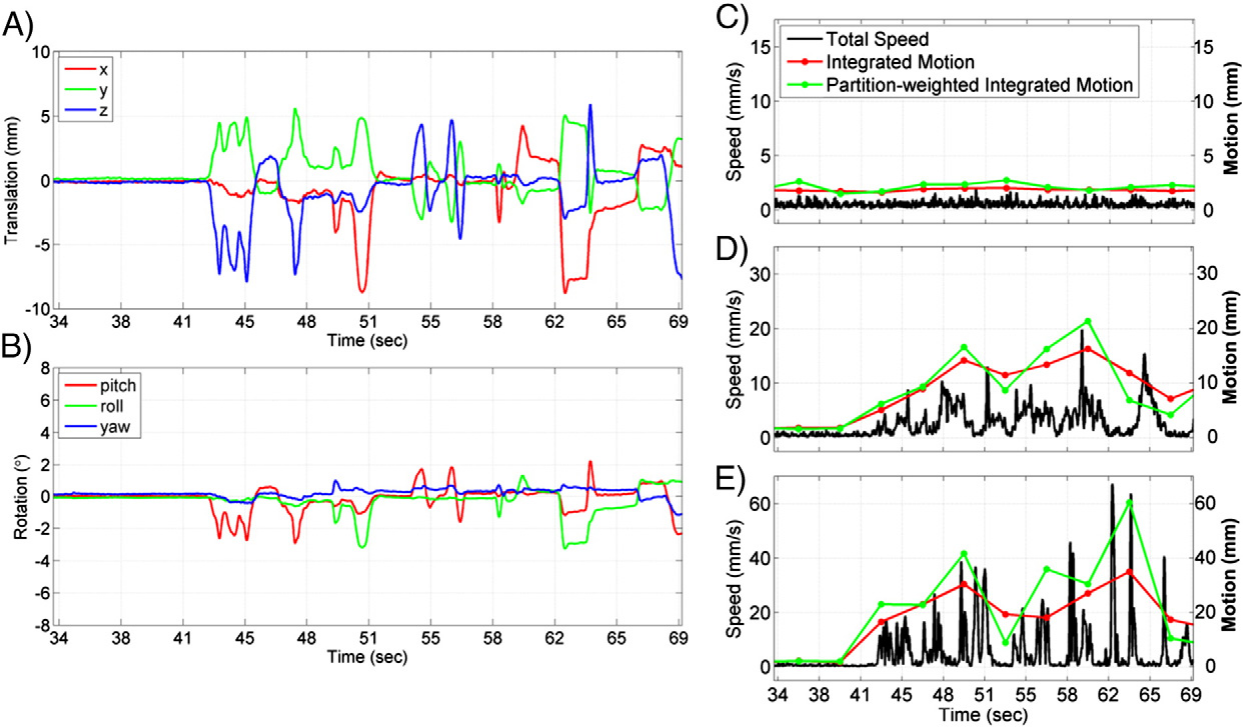
Example plots of motion data from Volunteer #2. The plots in panels A and B show the three translation and three rotation measurements from the optical camera (fast motion case). Panels C–E show the motion metrics calculated from the camera data for cases of no motion (C), slow motion (D), and fast motion (E, with data corresponding to plots in panels A and B). The grid lines indicate the timing of the MR volume acquisition. Note the different scale on the axes for panels C–E.

Fig. 2 shows an example of the motion data that the camera captures and how it is used to create the motion metrics. The three translation and three rotation measurements shown in Figs. 2A and B were from Volunteer #2 during experimental conditions of 1.5 mm resolution imaging, PMC off, and fast head motion. The amplitude of the movements shown in these plots was similar across volunteers. It is larger and more frequent than the type of motion typically seen in fMRI studies with healthy volunteers but roughly in line with the amplitude of motion seen in certain patient populations (Kochunov et al., 2006; Lemieux et al., 2007; Schulz et al., 2014; Versluis et al., 2010). Figs. 2C–E show the motion metrics of total speed, integrated motion, and partition-weighted integrated motion, all from Volunteer #2 with PMC off and no, slow, and fast motion respectively. The data shown in Fig. 2E corresponds to the data shown in Figs. 2A and B, giving an indication of how the metrics *M* and *M_PW_* are related to the translation and rotation measurements. The grid lines indicate the timing of the MR data acquisition per image volume and the dot markers on the motion metrics indicate the timing of the central k-space partition.

**Fig. 3:**
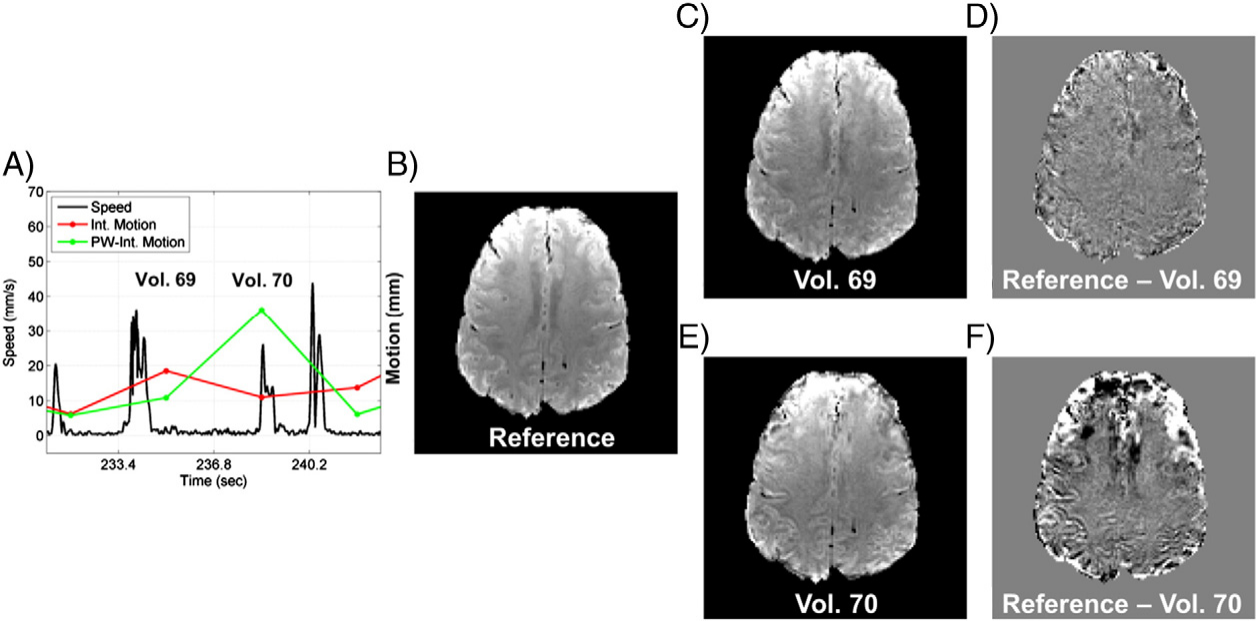
Example of how motion timing with respect to 3D data acquisition affects image quality. Panel A shows the motion metrics during the data acquisition of the 69th and 70th image volumes from Volunteer #2 (1.5 mm resolution, PMC off, fast motion), where the motion is occurring in the peripheral partitions for volume 69 and in the central partitions for volume 70. Panels B–E display one slice of the reconstructed volume, showing the reference image (B), the reconstructed volumes 69 and 70 (C and E), and the difference images (D and F).

Fig. 3 shows an example of the importance of motion timing with respect to k-space partition acquisition that motivates the *M_PW_* metric. Fig. 3A shows the motion metrics during two consecutive image volumes acquired from Volunteer #2 (1.5 mm resolution, PMC off, fast motion). More motion occurred during the acquisition of image volume 69, but the motion mainly occurred as the peripheral k-space partitions were being acquired, whereas the motion from image volume 70 was coincident with the acquisition of the central portion of k-space. Fig. 3B shows the baseline image from this run that was used as the motion-free reference image, and respective images and difference images from volumes 69 and 70 are shown in Figs. 2C–F. It can be seen that the image error was worse for image volume 70, which supports the *M_PW_* metric being a better predictor of the artifact level than the simple integrated motion metric.

Two measures of image quality were calculated to quantify the effect that motion had on the acquired data, the tSNR of the time series and the root mean square error (RMSE) of all individual image volumes. Before calculation of the metrics, all 4D image data sets were registered to their respective first image in the 100-volume time series using the Estimate & Reslice algorithm in SPM 8 (Ashburner and Friston, 2003; SPM8), and the first five of the 100 image volumes were discarded to allow for equilibration of the longitudinal magnetization. The high resolution scans had an initial period of “dummy” data acquisition to allow for longitudinal magnetization equilibration before acquisition of the calibration data for the GRAPPA kernel, but the first five volumes were still discarded in order to match the number of volumes kept in the low resolution scanning cases. The tSNR was calculated on a voxel-by-voxel basis as the mean signal over time divided by the temporal standard deviation of the signal. Before calculating the temporal standard deviation, a first order detrending was performed. The reference image for the RMSE calculation was obtained by averaging all image volumes in the time series that had an *M_PW_* value that was at or below the *M_PW_* values of the baseline images (before motion commenced). A 3D mask was created to cover the entire brain and exclude signal from the skin surface and background noise. The entire 4D data set was scaled by the mean signal of all voxels within this mask. An RMSE value was calculated for each image volume using the reference image and all voxels within the mask. While the tSNR values gave an indication of the quality of the image data over the entire time series, the RMSE metric reflected the image quality of individual image volumes as a function of the amount of motion that occurred during data acquisition.

The FOV coverage in the head–feet direction was different for the 3.0 mm and 1.5 mm cases. To facilitate comparison between the two types of scans, a common volume was chosen for the analysis that started several millimeters above the top of the brain and covered 48 mm towards the feet (16 slices for the 3.0 mm data and 32 slices for the 1.5 mm data).

The data sets from the task fMRI experiments were processed in a standard pipeline using SPM8 (Friston et al., 2007; SPM8). The first five image volumes were discarded to be consistent with the analysis of the task-free data. All image volumes from each data set were realigned to the first image but no spatial smoothing was performed in order to maintain the high resolution of the data. The general linear model used for fitting the data contained only the task indicators for left/right stimulation and a high pass filtering cut off of 128 s. To measure the BOLD sensitivity across the four conditions of the factorial design, t-scores were analyzed for all voxels within the respective regions of interest (ROI) covering the visual cortex and the motor and sensorimotor cortices for the visual and motor experiments, respectively.

## Results

### Task-free fMRI

**Fig. 4:**
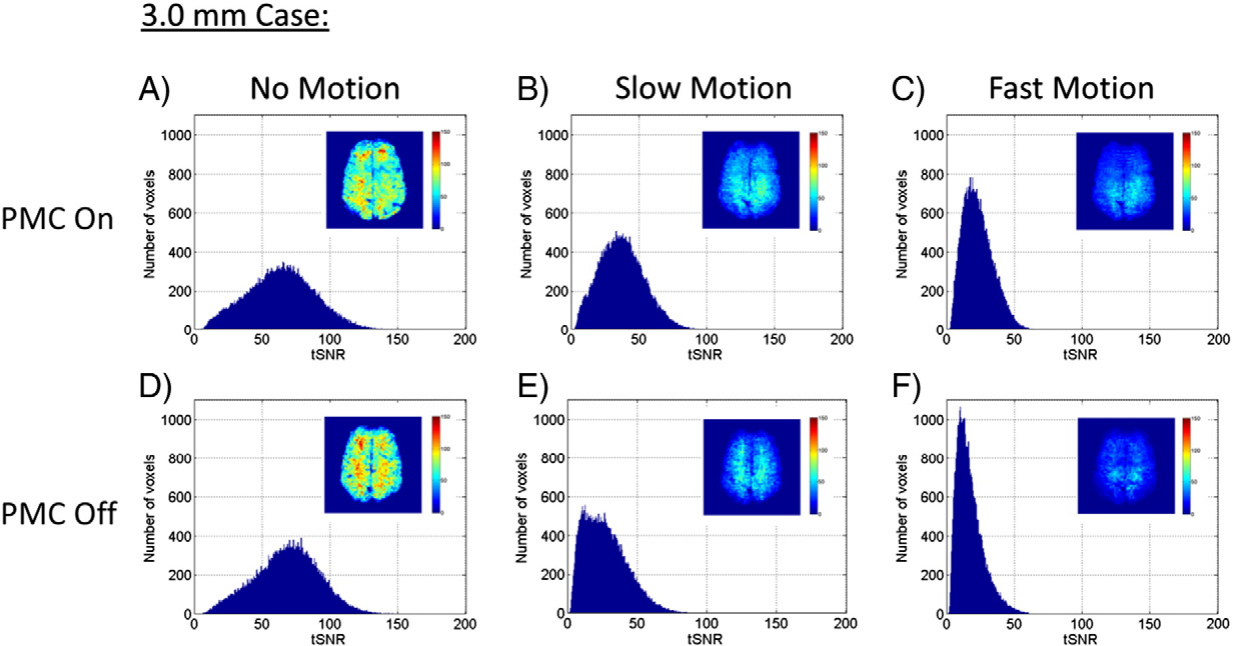
tSNR histograms for 3.0 mm data comparing PMC on vs PMC off for cases of no, slow, and fast motion. The tSNR values have been pooled from all five volunteers into one histogram for each condition. The inset image in each histogram shows one slice through the tSNR map from Volunteer #3.

**Fig. 5:**
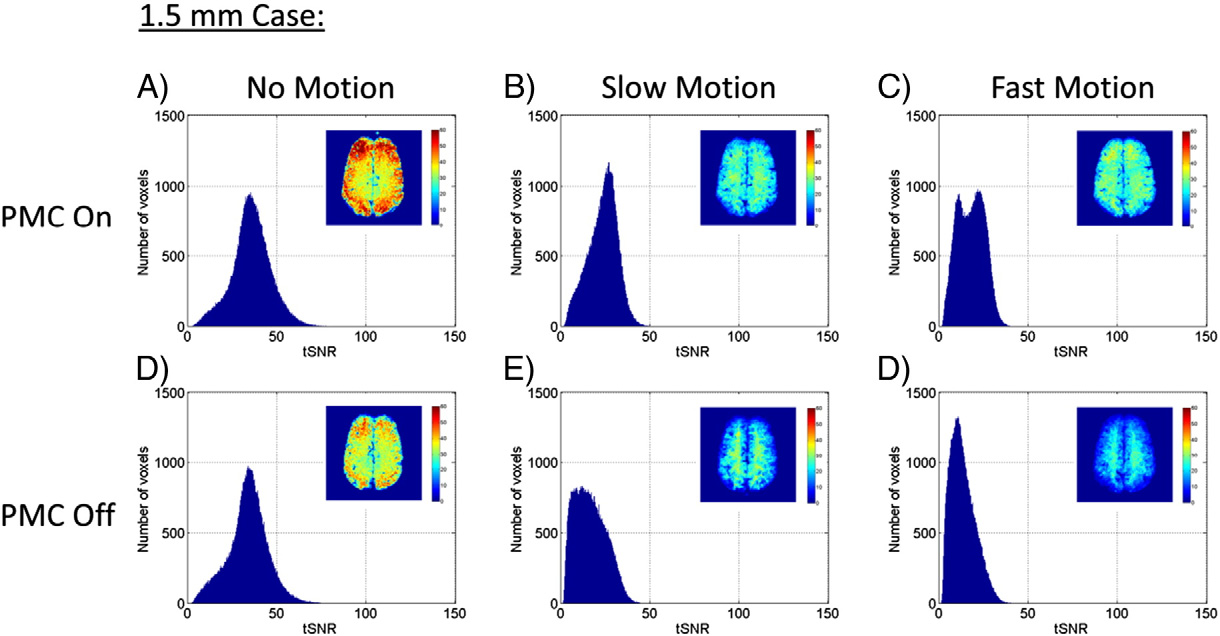
tSNR histograms for 1.5 mm data comparing PMC on vs PMC off for cases of no, slow, and fast motion. The tSNR values have been pooled from all five volunteers into one histogram for each condition. The inset image in each histogram shows one slice through the tSNR map from Volunteer #3. Note that the scales are different on both the histogram axes and tSNR maps from Fig. 4.

The tSNR results over all twelve experimental conditions are summarized in Figs. 4 and 5, and Table 1. Fig. 4 shows data from the 3.0 mm resolution runs, comparing the PMC on vs PMC off conditions for no motion, slow motion, and fast motion. The histograms of tSNR values use data that has been pooled over all five subjects. The example images in the upper right corner of each plot show one slice through the tSNR maps of Volunteer #3. The distribution of tSNR values were very similar for the no motion cases, indicating that the PMC system is not introducing any errors through spurious modifications of the RF pulses and imaging gradients. When motion was present, the tSNR distributions were shifted towards lower values, as would be expected. This occurred for both the PMC on and PMC off cases, but the average tSNR values for the slow and fast motion cases were 37% and 34% greater, respectively, for PMC on compared to PMC off.

Similar results are shown in Fig. 5 for the tSNR distributions from the 1.5 mm resolution runs, where the inset image of the tSNR map is also from Volunteer #3. Note that the scales on both the histogram axes and the tSNR maps are different from those in Fig. 4. The same effects as seen in the 3.0 mm resolution data were seen here as well: the no motion distributions of tSNR were very similar between PMC on and PMC off, the effect of decreasing tSNR with increasing motion was seen, and the runs with PMC on had clearly higher overall tSNR values for comparable levels of motion (43% improvement for the slow motion case, 32% improvement for the fast motion case). One slight anomaly is seen in the double-peaked distribution of the PMC on, fast motion case. This is due to the fact that Volunteer #5's movements during the fast motion cases were significantly greater than all of the other volunteer's movements, which created a subset of the pooled data that had significantly lower tSNR values. Volunteer #5 had consistently higher motion metrics for all fast motion cases, but the effect on the total distribution of tSNR values was not as obvious for the PMC off case or the 3.0 mm resolution cases.

The effects seen in the tSNR distributions are quantified in Table 1. The values are presented as the mean tSNR value and standard deviation over subjects. Two-tailed paired t-tests determined significant differences between the PMC on and PMC off conditions for all six combinations of the motion and resolution parameters. There was no significant difference between PMC on vs PMC off when there was no motion present (for either resolution), but the tSNR values for PMC on were significantly higher for all cases when motion was present. To check the levels of motion performed by the volunteers between the PMC on and PMC off cases, average values for the partition-weighted integrated motion metric are also reported in the parenthesis as the mean over all image volumes and standard deviation over subjects. Despite the fact that it was not possible to have the volunteers recreate exactly the same motion between the different runs, there were no significant differences in the average partition-weighted integrated motion values between the different paired PMC on and PMC off cases.

**Fig. 6:**
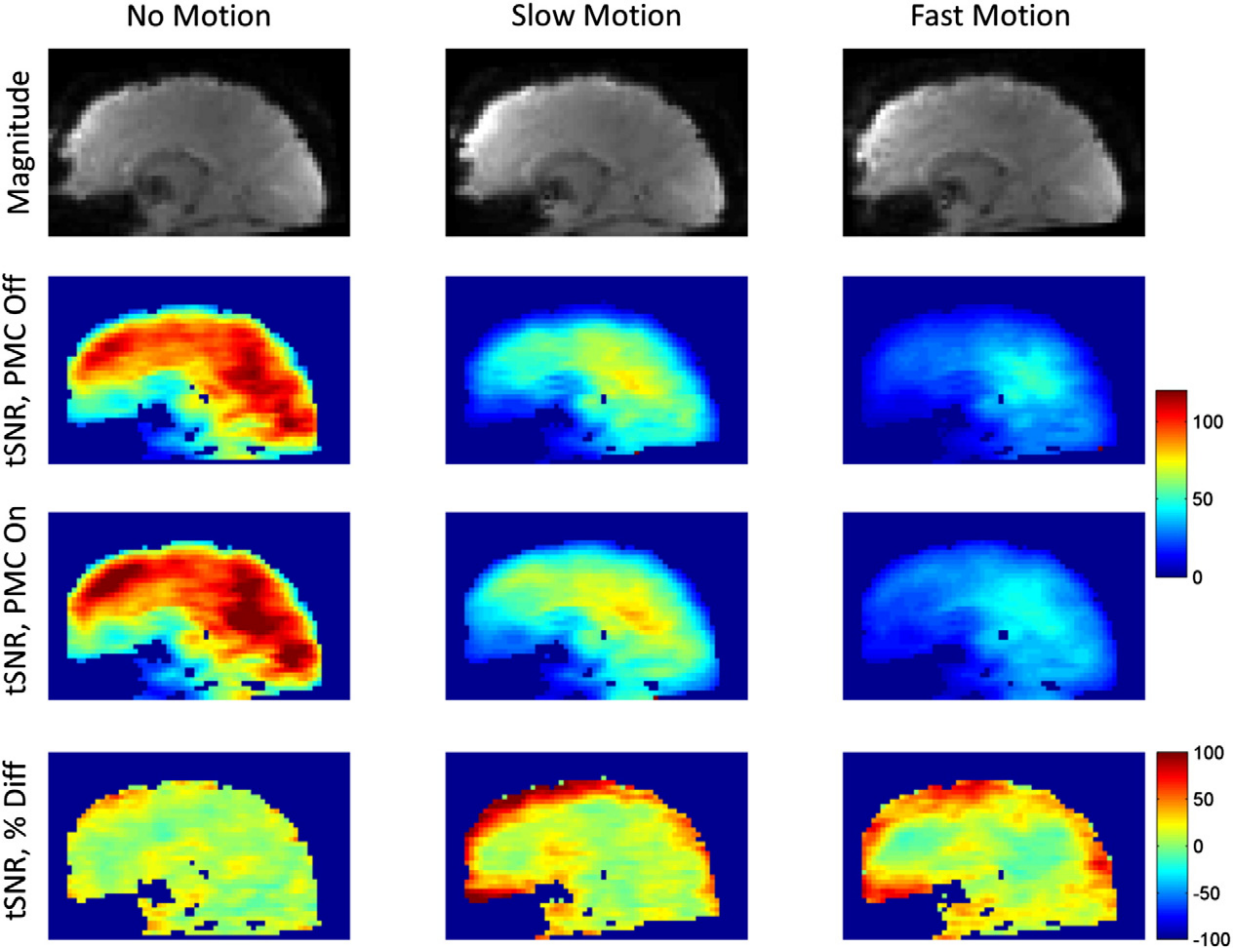
tSNR values averaged over all 5 volunteers, 3.0 mm isotropic data. The first row shows a sagittal slice of one representative magnitude image; the second and third rows show tSNR maps averaged over the five volunteers for the PMC off and PMC on cases; the fourth rows shows the percent difference between the tSNR values of the PMC off and PMC on cases ((PMC on − PMC off)/PMC off).

Fig. 6 illustrates how the losses in tSNR due to motion were distributed across the brain. The data are from the 3.0 mm resolution cases and averaged over all five volunteers. The first row displays sagittal slices of a representative magnitude image from the no motion, slow motion, and fast motion cases (all PMC off). The second and third rows show the average tSNR values for the PMC off and PMC on cases respectively. The fourth row displays the percent difference between the PMC on and PMC off cases, showing the spatial distribution of tSNR improvements that can be achieved by using PMC. For both the slow motion and fast motion cases, the largest improvements in tSNR occurred at the surface of the brain, while the tSNR values in many areas in the center of the brain did not show significant differences.

### Motion metrics and characterization

**Fig. 7:**
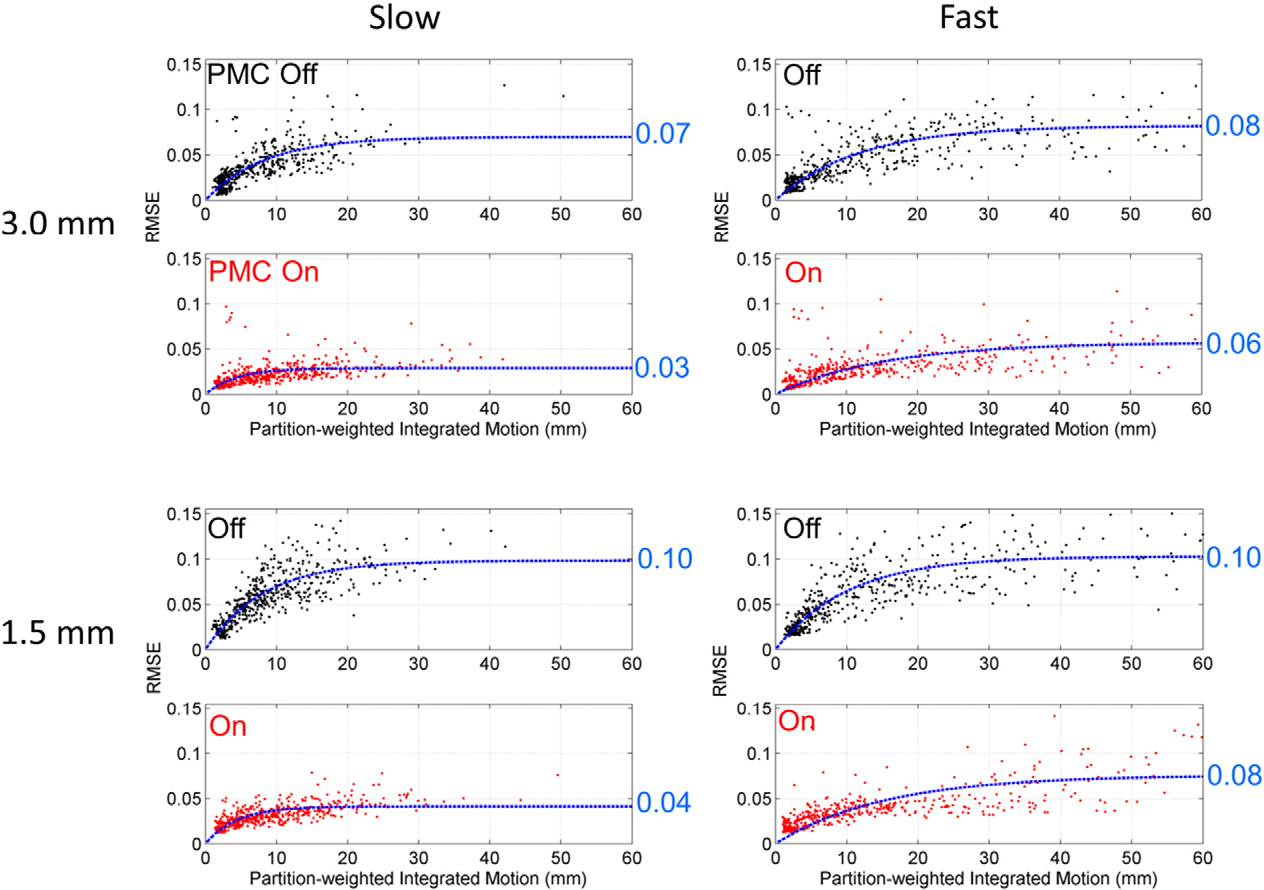
Scatter plots of RMSE vs partition-weighted integrated motion. Data from all image volumes and all volunteers are pooled for each of the cases shown. The blue dashed lines are fits of the data to the functional form of Eq. (5), with the blue text indicating the value of *A* from the fit.

While the tSNR values gave an indication of the quality of the image data over the entire time series of each run, the RMSE metric was intended to evaluate the image quality of individual image volumes as a function of the amount of motion that occurred during data acquisition. Fig. 7 presents scatter plots of the RMSE values against the partition-weighted integrated motion (*M_PW_*) values. The data were pooled over all subjects, with each dot representing one image volume. The blue dashed lines represent the fit to the function:

(5) $$\mathrm{RMSE}=A\cdot \left(1-\exp\left(-R\cdot M_{PW}\right)\right)\text{.}$$

where *A* and *R* are the fitted parameters. Several observations emerge from the scatter plots. The first is that RMSE increased with increasing values of *M_PW_*, as would be expected. However, the RMSE values did not increase linearly, but plateaued at some maximum level as *M_PW_* continued to increase. The fits to the data indicate that the RMSE values were, on average, lower for PMC on compared to PMC off for comparable values of *M_PW_*.

Goodness of fit tests were performed to evaluate the usefulness of the *M_PW_* metric compared to the simpler *M* metric for predicting motion-related artifacts. The function defined in Eq. (5) was fit using both the *M_PW_* and *M* values for the PMC off motion cases, and *r*^2^ values were calculated in all instances. The *r*^2^ values were higher for all four cases when *M_PW_* was used as the input. The range of *r*^2^ values was from 0.61 to 0.77 when using *M_PW_* compared to 0.51 to 0.68 when using *M*.

**Fig. 8:**
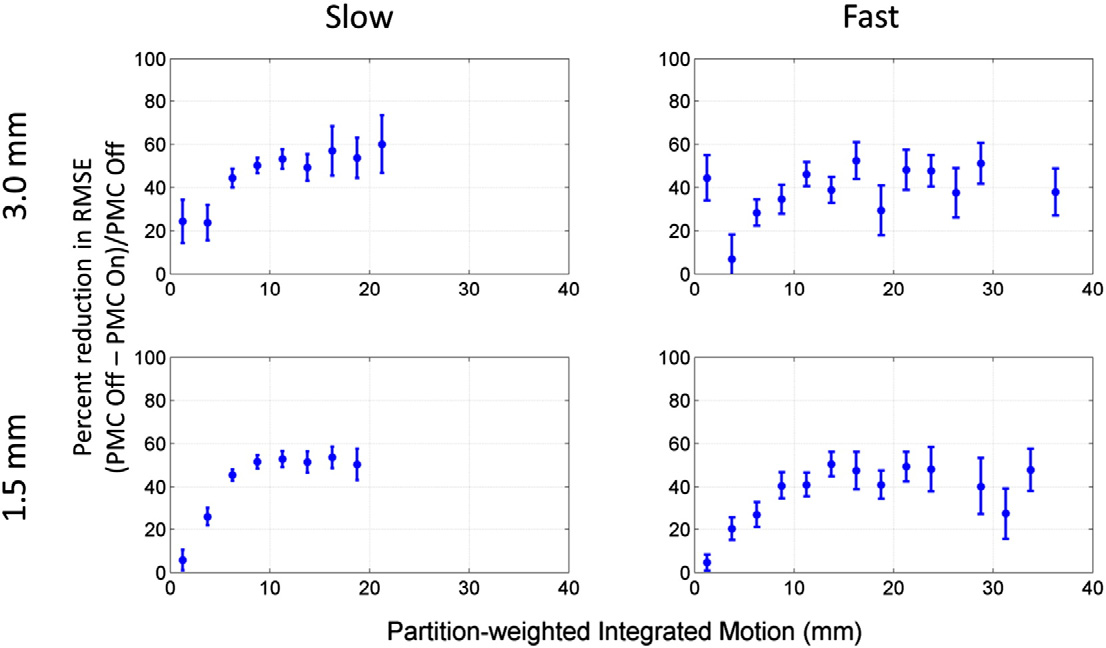
Plots of percent reduction in RMSE for PMC On compared to PMC Off as a function of partition-weighted integrated motion. The RMSE data presented in Fig. 7 were averaged over *M_PW_* bins that were 2.5 mm wide (only bins with 5 or more data points were used). The percent reduction in RMSE was calculated as (RMSE_PMC_Off_ − RMSE_PMC_On_) / RMSE_PMC_Off_. Error bars show standard error of the binned RMSE values propagated through the percent difference calculation.

Fig. 8 shows percent decrease in RMSE values from PMC off to PMC on cases as a function of *M_PW_*. The plots were generated by averaging the data shown in Fig. 7 over bins along the *M_PW_* dimension that were 2.5 mm wide. Only bins that had 5 or more data points were kept. The percent reduction in RMSE was calculated as (RMSE_PMC_Off_ − RMSE_PMC_On_) / RMSE_PMC_Off_, and the error bars show the standard errors of the binned RMSE values propagated through the percent difference calculation. The trend over the data sets showed small percent decreases in RMSE at low levels of motion (*M_PW_* < 5 mm), and then greater percent decreases in RMSE as the level of motion rises until a plateau was reached. This plateau in percent decrease of RMSE was larger for the slow motion cases (50%–60%) than for the fast motion cases (~ 40%).

### Task fMRI

**Fig. 9:**
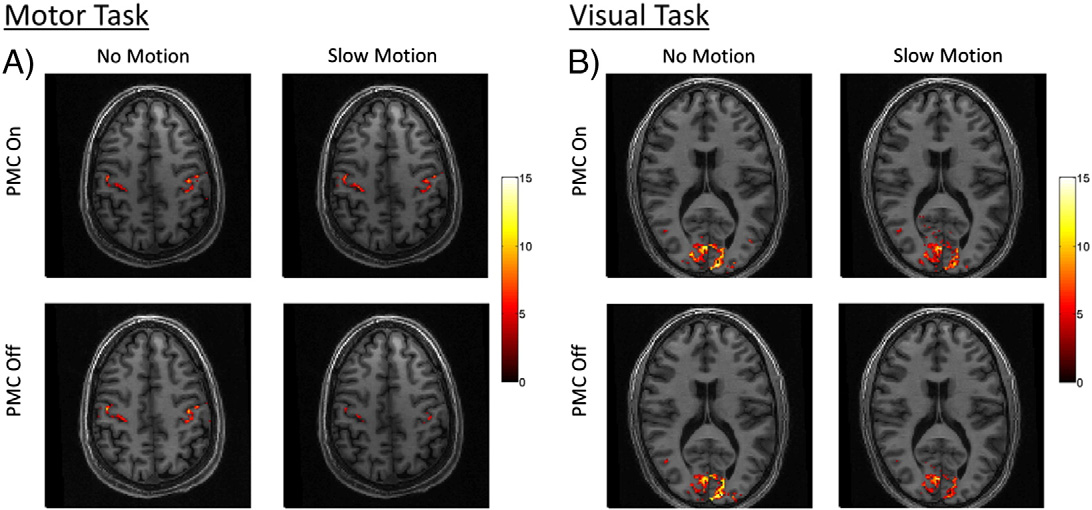
Statistical t-score maps of significantly activated voxels (uncorrected p < 0.001) from the task fMRI experiments. Results from 2 × 2 factorial design experiments showing combined activation maps from A) left and right finger-tapping stimulation and B) left and right visual hemi-field stimulation.

**Fig. 10:**
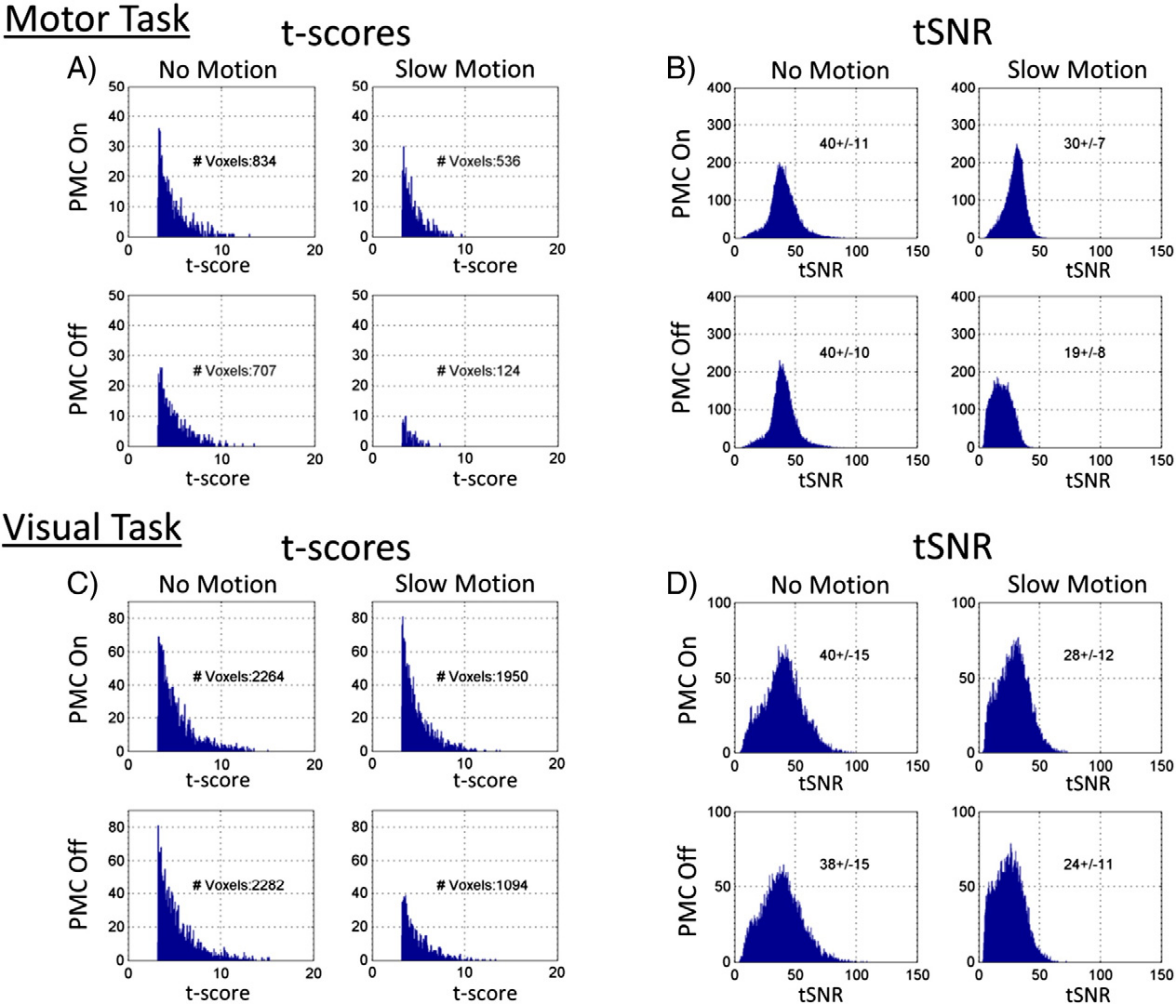
Task fMRI results. A) Histograms of motor task t-scores for PMC on vs PMC off and no motion vs motion cases. All voxels within an anatomically defined ROI having a t-score greater than 3.2 were included (corresponding to uncorrected p < 0.001). B) Histogram of tSNR values from all voxels within the same ROI. C) and D) T-score and tSNR histograms for the visual task.

The results from the two task fMRI experiments are shown in Figs. 9 and 10. Fig. 9 shows representative t-score maps of activated voxels (uncorrected p < 0.001) overlaid on a high resolution structural image for each of the four conditions of the 2 × 2 factorial design. The motor task results are displayed in Fig. 9A and the visual task results are displayed in Fig. 9B. For both tasks, the t-score maps indicate that the activation for the two no motion cases is very similar, but the activation appears significantly suppressed for the condition of slow motion and PMC off.

The task fMRI t-score and tSNR values are summarized in histograms in Fig. 10. The data shown were extracted from anatomically defined regions for the motor and visual cortices. The tSNR distributions from the four conditions show similar behavior as seen in the task-free fMRI results. When comparing the conditions of PMC on, no motion to PMC on, slow motion, the mean tSNR values decreased by 25% and 30%, for the motor and visual tasks respectively. The corresponding tSNR decreases for the PMC off cases were considerably larger, with percent decreases of 53% and 37%. The effect of these tSNR decreases on the BOLD sensitivity can be seen in the histograms of t-scores shown in Figs. 10A and C. The histograms show t-score values for all voxels that were within an anatomically defined region corresponding to where the activation is expected to occur and that had a t-score greater than 3.2 (corresponding to an uncorrected p < 0.001). For the no motion cases in both tasks, the t-score distributions had similar maximum t-score values and numbers of voxels activated. For the motion cases when PMC was on, the number of activated voxels decreased by 35% for the motor task and 14% for the visual task. When PMC was off, the drops in number of activated voxels were 82% and 52% for the two tasks.

## Discussion

The results presented here demonstrate that a PMC system using an external camera and marker with real-time feed-forward control to the imaging pulse sequence can significantly improve the data quality of 3D EPI images when motion occurs during data acquisition. For all conditions tested at different spatial resolutions and with differing amounts of deliberate head movement, the PMC system was able to improve the time series tSNR by 30% to 40% compared to uncorrected data. The tSNR increases translated into higher functional sensitivity in two separate fMRI test experiments. For the tested cases with no motion present, the PMC system was sufficiently precise and stable that its updates did not degrade the tSNR compared to the no correction cases. Multi-shot 3D EPI sequences are particularly susceptible to data degradation due to motion because of their relatively long acquisition time. Successfully integrating a PMC system to alleviate this problem represents a large step towards making 3D EPI sequences an attractive option for conducting fMRI studies.

The metrics developed for analyzing motion were designed to compress the very rich camera motion tracking data down into a lower dimensionality while maintaining enough information to make useful predictions about the effect of motion on the image quality. They have several advantages over previous motion assessment strategies that use the image data to retrospectively estimate the movement between scans (Lemieux et al., 2007; Wilke, 2012). The high frame rate of the optical camera (one sample every 12.5 ms) is fast enough to capture all rigid body physiological motion and allows for correlation of the motion with the timing of the data acquisition. For 2D imaging, motion can be correlated with the individually acquired slices, and for 3D imaging, as presented here, the motion can be correlated with the different partition encodings. Additionally, because the metrics are defined based on integrating the modulus time derivatives of the camera data, instead of the total displacement relative to some starting point, they can provide an accurate measure of how much total motion occurred throughout the data acquisition period. For example, a rapid nod that displaces the head significantly and then returns to nearly the original position might be calculated as only a small displacement using retrospective image-based approaches, but would be characterized by our metrics according to when the motion occurred relative to the partition acquisition timing. As another example, if the subject initially moves from their starting position but then remains still during the acquisition of subsequent image volumes, our metrics would calculate both the total speed and integrated motion metrics to be zero for these later volumes despite the displacement from the initial position. This is a purposeful feature of the metrics as no image artifact will be introduced in these volumes and the realignment to the original position can be well handled by post-processing methods.

The results shown in Fig. 7 indicate that the partition-weighted integrated motion metric for 3D EPI, *M_PW_*, is a good predictor of the level of artifact introduced into individual image volumes by motion when PMC is off. The scatter plots are noisy due to the complicated relationship between motion and image artifact, but a clear trend of increasing RMSE with increasing *M_PW_* can be seen. The functional form of Eq. (5) used to fit the data was not derived from physical principles of how motion affects k-space data acquisition, but was rather a heuristic model chosen because it fits the data well and reflects the fact that the RSME as a function of motion must eventually reach an asymptote. The results also show that incorporating information about the timing and relative weighting of the 3D k-space partitions allows for better correlation between the measured motion and resulting image artifacts. With some tailoring to the specific application, the motion quantification metrics presented here show enough predictive capability that they could be likely used as criteria in data rejection/resample schemes.

One problem faced by any study attempting to evaluate a motion correction scheme is how to best sample the infinite parameter space of possible motion trajectories. Subject motion seen during fMRI studies can vary according to factors such as the task being performed, whether the subjects are healthy or patients, which particular patient population the subjects are from, how securely the head is immobilized within the RF coil, if the subject is falling asleep during scanning, if the subject is coughing or swallowing in the scanner, and so on. This study design took the approach of instructing volunteers to perform a wide range of motion types that fell within constraints of what has been reported in the literature (Brown et al., 2010; Lemieux et al., 2007; Van Dijk et al., 2012), and then characterizing the level of motion present using the *M_PW_* metric. This approach has two advantages. First, it does not require that volunteers execute the exact same motion trajectory between “correction on” and “correction off” runs, which is a difficult task in practice. The level of motion present during “correction on” and “correction off” runs can be assessed with the *M_PW_* metric to determine if significant differences exist. Second, the performance of the motion correction scheme can be evaluated as a function of motion level. As Fig. 8 shows, the reduction in RMSE provided by the PMC system as a function of the motion level is not trivial. If only one type of motion had been performed, then only one point on this curve would be known. By performing a wide range of motion types, and characterizing them with the *M_PW_* metric, the entire curve is described.

The present study only evaluated the PMC system for the case of 3D EPI scanning. A direct extrapolation of these tSNR and t-score results to the more common approach of 2D EPI scanning for fMRI is not trivial given the different imaging parameters that would be used (e.g. longer TR and larger flip angles) and the fundamentally different data acquisition strategy. However, the implementation of the PMC system for 2D EPI scanning or 2D multiband EPI scanning (Setsompop et al., 2012) would be done in exactly the same way, with the imaging parameters still updated immediately before every RF pulse as shown by Schulz et al. (2014) and Speck et al. (2006). In addition to the benefits of rigid-body realignment discussed here, problems particular to 2D EPI scanning such as spin-history artifacts and intra-volume acquisition motion artifacts would also be expected to be improved. For both 3D EPI and 2D EPI scanning, the PMC system presented here can provide precise motion information to help avoid bias and false positives in the data analysis (Van Dijk et al., 2012) and may have the biggest impact for studies where larger levels of motion are unavoidable, such as overt speech studies (Price, 2012) or studies involving patient populations where non-compliance is a problem (Schlund et al., 2011).

Several other groups have implemented and evaluated similar fast prospective motion correction approaches for 2D EPI scanning for fMRI studies. Schulz et al. used a system consisting of three cameras tracking three passive optical markers for prospective motion correction during a leg movement task known to induce correlated head movements (Schulz et al., 2014, 2012). Their evaluation demonstrated that their PMC system was able to reduce the number of falsely activated voxels and improve the group level statistical power compared to the case of only doing volume-wise retrospective realignment. Rotenberg et al. implemented a scheme that combined prospective correction using a two-camera system tracking passive reflective markers with a real-time geometric distortion correction method (Rotenberg et al., 2013). Their results showed reduction of false activations during a finger-tapping task with cued intentional head movement. The approach presented by Muraskin et al. used active markers in their tracking system and demonstrated improved group-level statistics for block-designed flickering checkerboard, face localizer, and finger tapping paradigms (Muraskin et al., 2013).

While the PMC system shows great promise for reducing motion artifacts, practical limitations exist that may make the method unsuitable for certain fMRI studies. Perhaps the biggest practical issue is securely attaching the tracking marker to the subject's head. A number of approaches to fixating the marker to the subject's skin were tested, for example using an adhesive to attach it to the bridge of the nose or to the forehead, or attaching it to non-prescription eye glasses that the subject was wearing. However, each of these methods showed some degree of uncorrelated motion between the marker and the brain due to the malleability of the skin. Individually molded mini bite bars were therefore chosen for this study, but this may not be a feasible solution for all studies. For example, not all patient populations may tolerate the mini bite bars, it may be impractical to create many individual bite bars for a study involving a large number of subjects, or the mini bite bar set up may hinder certain speech related tasks.

The results in Table 1 show that the mean tSNR values decrease when motion occurs, even for the cases when PMC is on, indicating that the PMC system does not perfectly correct motion-induced errors. This is to be expected, as the PMC system only corrects for rigid body motion at discrete time points. This leaves many potential sources of error. While it is impossible to identify them all and separate out their individual contribution to the overall decrease in image quality, several major sources are known from previous studies (for an overview, see Maclaren et al., 2013). One of the biggest challenges is that motion continues to occur in between PMC updates. In this study, PMC updates were done every TR of 78 ms and maximum translation velocities along one dimension were typically in the range of 10–15 mm/s for the slow motion cases and 40–50 mm/s for the fast motion cases. Over the length of one TR, these values correspond to displacements of approximately 1 mm for the slow motion cases and 3 to 4 mm for the fast motion cases, which are clearly significant for the voxel sizes used. This long recognized problem could be ameliorated by performing PMC updates of the imaging gradients more often, as has been done by Herbst et al. (2012).

Other known contributors to motion-induced errors are related to the fact that the head is moving through inhomogeneous B0 and B1-receive fields, neither of which the PMC system controls. B0 field distortions due to susceptibility changes at tissue boundaries cause well known artifacts of geometric distortions and signal drop out in EPI (Hutton et al., 2013). Head movement will cause the level of these artifacts to change from volume to volume, leading to spurious changes in the signal over time. In addition to these B0 related effects, the head will also be moving through a static coil sensitivity profile that is highly inhomogeneous for modern multi-coil receiver arrays. Therefore, even if perfect rigid body realignment is achieved to correct inter-volume motion, the MR signal will still change due to modulation by the coil sensitivity profile. Internal experiments (not shown) carried out at our institution on phantoms undergoing step-wise motion in the head/foot direction with PMC on indicate that the signal can change by 1–2%/mm when imaged with the Siemens 32 channel head coil used in this study.

Finally, remaining sources of motion-induced error that pose a challenge for the PMC system include non-rigid body motion that can occur, for example, near the lower brainstem or cervical spinal cord (Mohammadi et al., 2012); signal changes that arise when movement changes the bulk susceptibility field (Hutton et al., 2013); signal fluctuations due to physiological noise (Hutton et al., 2011); and any latency that may occur between skull movement and brain movement. The problems of signal changes from susceptibility effects and physiological noise can be addressed using strategies that are complementary to the PMC system (Glover et al., 2000; Hutton et al., 2013; Lutti et al., 2013). However, the remaining problems related to motion will require a more sophisticated approach for full correction.

## Conclusion

Motion during fMRI studies is a persistent problem that is only partially addressed by standard retrospective image-based correction methods. The PMC system used here represents a further step forward due to its ability to pre-emptively suppress motion artifacts and more accurately characterize head motion. For a 3D EPI implementation, we have demonstrated temporal SNR improvements in the range of 30% to 40% and improvements in the numbers of significantly activated voxels during task fMRI in the range of 70% to 330%. We developed motion quantification metrics that reliably predict image artifact levels due to motion during data acquisition. The methods presented here can be directly extended to all types of fMRI studies, resting state or task-based with 2D EPI or 3D EPI scanning.

## Figures and Tables

**Table 1:** Comparison of tSNR values across all twelve experimental conditions. The mean tSNR values are reported with the standard deviation being over volunteers. Significant differences between the PMC on and PMC off cases are indicated by * for p < 0.05 and ** for p < 0.01. Mean and standard deviation of the partition-weighted integrated motion values are shown in parenthesis. The partition-weighted integrated motion values were not significantly different for any of the PMC On vs PMC off cases.

tSNR (partition-weighed integrated motion)	No motion	Slow motion	Fast motion
*3.0 mm case*			
PMC on	68.0 ± 2.9 (1.9 ± 0.3)	37.6 ± 6.6* (8.4 ± 1.9)	23.3 ± 6.5* (16.0 ± 9.0)
PMC off	63.6 ± 8.4 (2.3 ± 0.4)	27.3 ± 4.4 (6.5 ± 1.8)	17.4 ± 4.5 (15.3 ± 7.2)

*1.5 mm case*			
PMC on	35.5 ± 4.3 (2.5 ± 0.7)	23.7 ± 2.5** (8.1 ± 1.0)	18.0 ± 4.6* (14.3 ± 9.0)
PMC off	33.3 ± 3.9 (1.8 ± 0.2)	16.6 ± 2.9 (8.2 ± 1.8)	13.6 ± 3.0 (14.5 ± 6.7)
